# Adequate symptom relief justifies hepatic resection for benign disease

**DOI:** 10.1186/1471-2482-5-7

**Published:** 2005-04-01

**Authors:** Bram Fioole, Marike Kokke, Richard van Hillegersberg, Inne HM Borel Rinkes

**Affiliations:** 1 Department of Surgery (G.04.228), University Medical Centre Utrecht, PO Box 85500, 3508 GA Utrecht, The Netherlands

## Abstract

**Background:**

The purpose of this study was to evaluate the long-term results of partial liver resection for benign liver lesions.

**Methods:**

All patients operated on for benign liver lesions from 1991 to 2002 were included. Information was retrieved from medical records, the hospital registration system and by a telephonic questionnaire.

**Results:**

Twenty-eight patients with a median age of 41 years (17–71) were operated on (M/F ratio 5/23). The diagnosis was haemangioma in 8 patients, FNH in 6, HCA in 13 and angiomyolipoma in 1. Eight patients were known to have relevant co-morbidity. Median operating time was 207 minutes (45–360). The morbidity rate was 25% and no postoperative mortality was observed. Twenty-two patients (79%) had symptoms (mainly abdominal pain) prior to surgery. Twenty-five patients were reached for a questionnaire. The median follow up was 55 months (4–150). In 89% of patients preoperative symptoms had decreased or disappeared after surgery. Four patients developed late complications.

**Conclusion:**

Long-term follow up after liver surgery for benign liver lesions shows considerable symptom relief and patient satisfaction. In addition to a correct indication these results justify major surgery with associated morbidity and mortality.

## Background

Partial liver resection is an accepted treatment for primary and secondary malignancies of the liver. In experienced hands this operation is associated with mortality rates of less than 5% and morbidity of approximately 30% [1-4].

Unlike malignant liver tumours, the indication for resection of benign hepatic lesions, including haemangiomas, focal nodular hyperplasia (FNH) and hepatocellular adenomas (HCA) remains controversial [5-7]. Indications for resection of benign liver masses include: 1) severe or progressive symptoms, 2) uncertain diagnosis with a suspicion for malignancy, and 3) risk of haemorrhage or rupture. If possible, it is important to discern whether the presenting symptoms are due to the liver lesion detected, before proceeding with surgical intervention. Several studies have reported about the indication for surgery in various benign hepatic tumours. However, less is known about the long-term results of surgical treatment, particularly regarding symptom relief.

The present study was undertaken to evaluate the long-term results of partial liver resections for benign liver lesions, with emphasis on the course of symptomatology, long-term complication rate, and patient satisfaction.

## Methods

All patients treated by partial liver resection for benign lesions in the University Medical Centre Utrecht between January 1991 and December 2002 were included. Information about these patients was retrieved from medical records and the hospital registration system.

Preoperative parameters consisted of age, sex, diagnosis, co-morbidity, presenting symptoms and indication for resection. In the preoperative work up we have routinely performed physical diagnostic investigation, ultrasound and computed tomography (CT) to exclude other pathology causing the symptoms. On indication additional gastroscopy or endoscopic retrograde cholangiopancreatography was performed. The indications for resection of a haemangioma were persisting symptoms and rapid growth. After exclusion of other pain aetiology a period of 3 months observation was allowed to asses the persistence of the symptoms. In case of FNH the indications for resection were persisting symptoms and to exclude malignancy. HCA's were resected if symptomatic or larger than 5 cm.

Date of resection, extent of resection, number of perioperative blood transfusions and duration of resection were considered perioperative parameters. A major resection was defined as a resection of three segments or more. Perioperative blood transfusion was defined as at least one unit of packed cells infused within 24 hours after surgery.

Documented postoperative parameters consisted of clinically relevant complications, postoperative mortality, duration of admission and follow up. Postoperative mortality was defined as in-hospital mortality. Long-term follow up was obtained by a telephonic questionnaire. Information was collected about the presenting symptoms, the relief of these symptoms after surgery and the impact on physical and social activities. The limitations on physical and social activities as a result of the presenting symptoms were divided in severe, moderate and none. Moderate limitations were defined as limitations for activities at least once a week, while severely limited patients experienced daily restrictions. Symptom relief was defined as a decrease or absence of the presenting symptoms after surgery.

## Results

A total of 28 patients were operated on for benign liver lesions. The diagnosis was a haemangioma in 8 patients (29%), FNH in 6 (21%), HCA in 13 (46%) and angiomyolipoma in 1 (4%). The group consisted of 23 female and 5 male patients with a median age of 41 years (range 17–71). Eight patients (29%) were known to have relevant co-morbidity, including severe cardiac or pulmonary diseases, diabetes mellitus, hepatitis, liver fibrosis, adipositas and multiple sclerosis.

Twenty-two patients were known to have symptoms prior to surgery (79%). The most frequent presenting symptom was upper abdominal pain (64%). Other presenting symptoms are shown in table 1. The most important indications for resection were symptoms and excluding malignancy (table 2). The symptoms mentioned in this table consisted of abdominal pain in all patients. Three patients with HCA presented with haemorrhage as a result of spontaneous rupture. One patient was immediately operated on. The other 2 patients were stabilized and resection was performed after the haematoma was resolved.

**Table 1:** Predominant presenting symptoms in 28 patients

Symptom	Haemangioma (n = 8)	FNH (n = 6)	HCA (n = 13)	Angiomyolipoma (n = 1)	Total (n = 28)
Abdominal pain	7	4	7	0	18
Swelling	1	1	0	1	3
Nausea	0	0	1	0	1
No symptoms	0	1	5	0	6

**Table 2:** Indication for resection in 28 patients

Indication	Haemangioma (n = 8)	FNH (n = 6)	HCA (n = 13)	Angiomyolipoma (n = 1)	Total (n = 28)
Symptoms	7	1	3	0	11
Excluding malignancy	0	5	5	1	11
Haemorrhage	0	0	3	0	3
Risk of malignant degeneration	0	0	2	0	2
Size	1	0	0	0	1

All operations concerned partial liver resections, of which 11 resections were major (39%) (Table 3). The median operating time was 207 minutes (range 45–360). Eighteen procedures necessitated perioperative blood transfusions (median 2, range 0–16).

**Table 3:** 28 Partial liver resections

		Extent of resection			
Diagnosis	Patients	L Hemi	R Hemi	Ext R	Segm

Haemangioma	8	1	1	1	5
FNH	6	0	2	0	4
HCA	13	0	4	0	9*
Angiomyolipoma	1	1	0	0	0
All	28	2	7	1	18

L Hemi = Left hemihepatectomy

R Hemi = Right hemihepatectomy

R Ext = Extended right hemihepatectomy

Segm = Segmentectomy

* One patient treated with a central resection segment 4, 5 and 8

One patient underwent extended left hemihepatectomy for an asymptomatic, but rapidly growing giant haemangioma (25 cm). This major resection was accompanied by massive intraoperative haemorrhage from direct venous hepatocaval branches (7500 ml). After the resection the transected surface kept oozing. Therefore haemostasis was obtained by packing with gauzes. About 36 hours later the gauzes were removed. Further postoperative recovery was uneventful. A second patient underwent reoperation for wound dehiscence. The median hospital stay was 9.5 days (range 5–39). Seven patients developed one or more postoperative complications resulting in a morbidity rate of 25% (table 4). No postoperative mortality was observed.

**Table 4:** Seven patients with 8 postoperative complications

Complication	Patients
Wound infection	2
Urinary tract infection	2
Bile leakage	1
Severe ascites	1
Pneumonia	1
Deep venous thrombosis	1

Total	8

**Figure 1:**
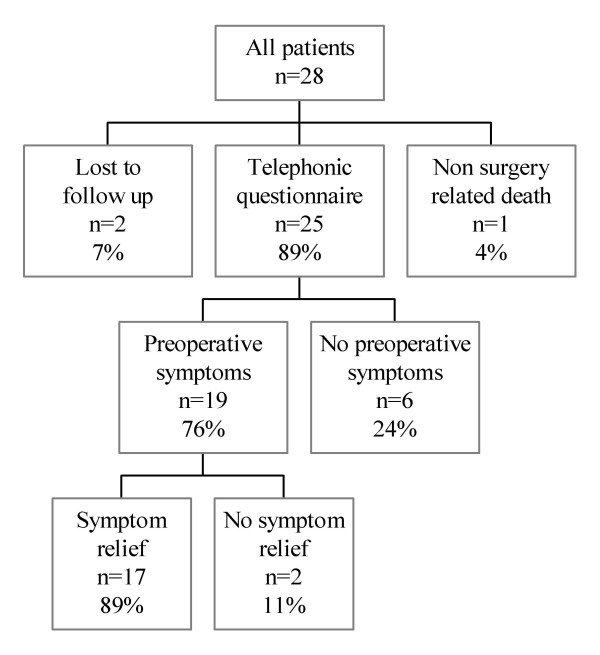
Patients summary

During follow up one patient died as a result of a cause other than liver surgery. Three months after a left hemihepatectomy for a large tumour which turned out to be a angiomyolipoma this patient died of cerebral stroke. Of the remaining 27 patients we were able to contact 25 for a questionnaire (figure 1). The median follow up of the interviewed patients was 55 months (range 4–150). Six of the 25 interviewed patients had no symptoms prior to surgery and underwent resection because of an uncertain diagnosis of an incidentally discovered liver lesion. Before surgery 7 patients were severely limited in their physical activities as a result of the liver lesion, 5 were moderately limited and 13 were not limited. Considering social activities 4 patients were severely limited prior to surgery, 4 were moderately limited and 17 were not limited. After surgery the symptoms had decreased or disappeared in 17 of the 19 interviewed patients with preoperative symptoms (89%). In two patients the symptoms were unchanged. One patient underwent partial liver resection for an adenoma. After the resection the preoperative abdominal pain never decreased. In a second patient preoperative abdominal pain did not decrease after resection of a large haemangioma. In these two cases the preoperative symptoms were probably not related to the liver lesion. Four of the 25 interviewed patients had developed late complications as a result of the operation. These complications consisted of a hypertrophic scar (n = 2) and incisional hernia (n = 2). Twenty-three of 25 interviewed patients were satisfied with the result of the resection (92%).

## Discussion

Symptom relief was observed in 89% of the patients (17/19), while in two patients the preoperative symptoms had not decreased. Only a few studies concerning long-term follow up of resections for benign liver tumours have been published. Terkivatan et al. described 80% symptom relief in surgically treated patients [5]. In this study surgery was only indicated in case of suspicion of malignancy, severe or increasing symptoms or a HCA larger than 5 cm. They compared this group retrospectively with patients treated conservatively, of whom 34% presented with symptoms, e.g. non-specific complaints of fatigue and mild abdominal pain considered unrelated to the tumour. During long-term follow up 87% symptom relief was observed in the group who was treated conservatively. Charny et al. registered 93% symptom regression in patients who underwent partial liver resection for benign liver tumours and 86% symptom regression in patients who were observed for symptoms considered unrelated to the tumour or treated for unrelated conditions [8]. Therefore partial liver resection for a symptomatic liver lesion should only be performed when symptoms are most likely related to the lesion.

Because of the nature of benign liver tumours, clear indications are needed for partial liver resection, an operation associated with substantial postoperative morbidity and mortality. Indications for resection of a cavernous haemangioma of the liver are the development of complications, rapid growth, the presence of persisting symptoms or the need to establish a confident diagnosis. The potential for complications of a liver haemangioma (mainly rupture) is not an indication for resection of all liver haemangiomas. Spontaneous rupture of a haemangioma is infrequent and could be controlled with transcatheter hepatic artery embolization prior to resection [9]. As for rapid growth, we have operated on 1 patient with an asymptomatic, but rapidly growing haemangioma in our series. Little is known about the natural history of these large haemangiomas and resection can be very challenging, since they are at risk for massive intraoperative haemorrhage. In case of abdominal pain the main challenge is to determine whether these symptoms are due to the haemangioma or an associated condition [10-12]. Farges et al. described other pain aetiology in 54% of patients with symptomatic haemangiomas [7]. Gandolfi et al. observed only 7% symptomatic giant haemangiomas in their series [13]. Haemangiomas are not likely to cause diagnostic uncertainty. In our series we have not performed diagnostic resections for haemangiomas.

Unlike FNH, HCA is often symptomatic and is noted for its spontaneous rupture and malignant transformation. Looking at the potential complications, HCA's with a diameter of more than 5 cm should be resected, while for smaller HCA's and FNH observation is justifiable [6,14-16]. Increasing size on radiographic imaging during observation is an indication for resection. FNH and HCA occur predominantly in females and are associated with long-term contraceptive steroid use [17]. This medication should be stopped, when FNH or a small HCA is not treated surgically. In case of FNH and HCA the most frequent indication for resection is the uncertain diagnosis of a hepatic mass with suspicion for malignancy. In addition to ultrasound, CT and MRI, positron emission tomography has proved to be a helpful modality distinguishing between benign and malignant liver lesions [18,19]. On the other hand, the use of needle biopsy should be reserved for atypical cases, since the limited material is rarely sufficient to exclude malignancy. In our series diagnostic uncertainty accounted for operation in 11 of 20 resected patients and was, together with symptoms, the main indication for resection. The final diagnosis after pathological examination of resected specimens was FNH in 5 patients, HCA in 5 and angiomyolipoma in 1. All HCA's were larger than 5 cm, but no malignancies were observed.

## Conclusion

We have shown in this consecutive series that partial liver resection for benign disease is a very effective procedure to relief invalidating abdominal symptoms. Benign liver lesions should only be resected when symptoms are most likely related to the lesion. In experienced centres the resection can be performed with acceptable morbidity and low mortality.

## Competing interests

The author(s) declare that they have no competing interests.

## Authors' contributions

BF designed the study, helped with the data acquisition and drafted the manuscript. MK carried out the data acquisition and participated in the study design. RH participated in the design of the study and in drafting and revising the manuscript. IBR conceived of the study and coordinated the draft and revision of the manuscript. All authors read and approved the final manuscript.

## Pre-publication history

The pre-publication history for this paper can be accessed here:


